# A New Wood Adhesive Based on Recycling *Camellia oleifera* Cake-Protein: Preparation and Properties

**DOI:** 10.3390/ma15051659

**Published:** 2022-02-23

**Authors:** Xue Deng, Zhigang Wu, Bengang Zhang, Hong Lei, Jiankun Liang, Lifen Li, Yuan Tu, De Li, Guoming Xiao

**Affiliations:** 1 College of Forestry, Guizhou University, Guiyang 550025, China; xue1208776758@163.com (X.D.); lifenli2011@163.com (L.L.); ty0822006x@163.com (Y.T.); lide228@163.com (D.L.); xgm17685381639@163.com (G.X.); 2 Yunnan Provincial Key Laboratory of Wood Adhesives and Glued Products, Southwest Forestry University, Kunming 650224, China; zbg18082968142@163.com; 3 College of Civil Engineering, Kaili University, Qiandongnan 556011, China; dushimensheng@126.com

**Keywords:** *Camellia oleifera*, cake protein, wood adhesive, crosslinking, bonding performance

## Abstract

In order to improve the initial viscosity and stability of *Camellia oleifera* cake-protein adhesive, *Camellia oleifera* cake-protein was blended with defatted soybean protein (DSP), soybean protein isolate (SPI), and casein, followed by adhesive preparation through degradation and crosslinking methods. The performance of *Camellia oleifera* cake-protein adhesive was investigated by Fourier transform infrared spectroscopy (FT-IR), differential scanning calorimetry (DSC), scanning electron microscopic (SEM), and thermogravimetric (TG) and X-ray diffraction (XRD). The results showed that DSP, SPI, and casein likely promoted the effective degradation of *Camellia oleifera* cake-protein, and, thus, more active groups were formed in the system, accompanied by more reactivity sites. The prepared adhesive had a lower curing temperature, and higher initial viscosity and stability, but the storage time was shortened. Moreover, DSP, SPI, and casein, themselves, were degraded into peptide chains with lower molecular weights; thus, improving the overall flexibility of the adhesive, facilitating a better elastic contact and regular array between crosslinking products, and further strengthening the crosslinked structure and density of the products. After curing, a compact and coherent reticular structure was formed in the adhesive layer, with both bonding strength and water resistance being significantly improved. According to the results obtained, the next step will be to study the DSP-modified *Camellia oleifera* cake-protein adhesive in depth.

## 1. Introduction

Wood adhesives are key to the development of the wood-based panel industry and a breakthrough point for manufacturing new products and making progress in the wood-based panel industry [1,2,3,4]. Phenolic formaldehyde resins, urea formaldehyde resins, and melamine-urea-formaldehyde resins are widely applied in the traditional wood industry. Their preparation, use as well as the processing and use of the panels release formaldehyde, endangering the environment and human health [5,6,7,8,9]. With the increasing shortage of global petroleum resources and gradually increasing environmental awareness, attention has increasingly turned toward renewable biomass resources. Preparing biomass wood adhesives with natural substances as raw materials (e.g., starch, lignin, tannin, and soy protein) has become a current research hotspot and development trend in the field of wood adhesives, among which soy protein-based adhesives have been extensively explored [10,11,12,13,14,15,16]. At present, soy protein-based adhesives have developed into mainstream biomass wood adhesives, where various soy protein adhesives have been industrially applied to plywoods and laminated core boards [17,18,19,20]. During the 13th Five-Year Plan period in China, major breakthroughs were made in key technologies of biomass adhesives, represented by soy protein-based adhesives, and the consumption of biomass adhesives increased rapidly. In 2018, the annual consumption of soy protein-based adhesives in China was about 25,000 tons, up 67% year-on-year, and they were mainly used for the preparation of plywood. However, the large-scale application of soybean, which is an important food crop, as the raw material for wood adhesives will affect the food security of human beings. Hence, it is especially important to look for a resource ‘not fighting against humans for grain or against grain for land’ to prepare protein adhesives.

Pizzi et al. [21,22] and Guo et al. [23] pointed out that a large number of oilcakeby-products rich in proteins and carbohydrates and generated in the refining process of edible oil and biodiesel have the characteristics and potential for preparing wood adhesives. At present, there are few studies on the preparation of wood adhesives with oil cakes. The performance of cold-pressed walnut-cake protein adhesives prepared by Wang et al. met the strength requirement of Type III plywood in Chinese National Standard GB/T9846—2015 [24]; that of cold-pressed sesame cake-protein adhesives prepared by Wei et al. satisfied the use requirement of Type II plywood in Chinese National Standard GB/T9846—2015 [25]; and that of peanut-cake protein adhesives prepared by Chen et al. achieved the use requirement of Type I plywood in Chinese National Standard GB/T9846—2015 [26]. The preparation of wood adhesives with *Jatropha curcas*-cake protein has been extensively investigated. As indicated by Lestari et al. [27], *Jatropha curcas*-cake has considerable potential for foaming, paper adhesives, and wood adhesives. The results of Zhang et al. showed that the bonding performance of *Jatropha curcas* cake-protein adhesive was very significantly influenced by pH, the curing temperature of the modified adhesive was obviously lowered, and the bonding strength of plywood made of this adhesive was evidently improved [28]. Hamarneh et al. pointed out that the bonding strength of polyketone-based adhesives could be substantially enhanced by *Jatropha curcas* cake-protein [29]. Chi suggested that *Jatropha curcas* cake-protein adhesive differs little from soy protein-based adhesive in bonding strength, but its water resistance was stronger than soy protein-based adhesive [30]. Wang et al. revealed in their study that *Jatropha curcas* cake-protein adhesive, rubber cake-protein adhesive, and soybean protein adhesive showed similar curing characteristics [31].

In the previous research, it was discovered that the initial viscosity and storage stability of the wood adhesive prepared using *Camellia oleifera* cake-protein were poor [32,33]. Therefore, in this study, *Camellia oleifera* cake-protein was blended with defatted soybean protein (DSP), soybean protein isolate (SPI), and casein, expecting to improve the initial viscosity and storage stability of *Camellia oleifera* cake-protein adhesive, and, moreover, to enhance its bonding performance and water resistance.

## 2. Materials and Methods

### 2.1. Materials

*Camellia oleifera* cake-protein (protein content 36%) was obtained by processing *Camellia oleifera* cakes from Ruizhaoyuan Co., Ltd. (Rongjiang, China). Defatted soybean protein (DSP, protein content 53.4%) was purchased from Shandong Scents Soy Protein Co., Ltd. (Binzhou, China). Soybean protein isolate (SPI, protein content 90%) and casein (protein content 92%) were bought from Shandong Gushen Biotechnology Group Co., Ltd. (Dezhou, China). The epoxy was purchased from the market, and the epoxide value and softening point of E-44 epoxy resin were 0.41–0.47 and 12–20 °C, respectively. In addition, *Populus* spp. veneer with a length × width of 400 mm × 400 mm, thickness of 2 mm, and moisture content of 8–10% was purchased from Qunyou Wood Co., Ltd., Suzhou, China. The other chemical reagents from Sinopharm Chemical Reagent Co., Ltd. (Shanghai, China) such as NaOH and urea were all analytically pure.

### 2.2. Preparation of Camellia oleifera Cake-Protein Hydrolysates

Preparation of pure *Camellia oleifera* cake-protein hydrolysate: 200 g of water was added to a round-bottom three-mouth flask equipped with a mechanical stirring rod, thermometer, and condenser pipe. Next, mechanical stirring was started, 80 g of *Camellia oleifera* cake-protein and 1.2 g of sodium dodecyl benzene sulfonate (SDBS) were added, and then the mixture was heated to 65 °C. After that, 4.8 g of sodium hydroxide was added and kept for 90 min, and then 8 g of urea was added and kept for 20 min, followed by cooling and emptying.

Preparation of *Camellia oleifera* cake-protein compound hydrolysate: 200 g of water was added to a round-bottom three-mouth flask equipped with a mechanical stirring rod, thermometer, and condenser pipe. Next, mechanical stirring was started, and 72 g of *Camellia oleifera* cake-protein, 8 g of DSP, and 1.2 g of SDBS were added. After the mixture was heated to 65 °C, 4.8 g of sodium hydroxide was added and kept for 90 min, and then 8 g of urea was added and kept for 20 min, followed by cooling and emptying. In this way, DSP/*Camellia oleifera* cake-protein compound hydrolysate, SPI/*Camellia oleifera* cake-protein compound hydrolysate, and casein/*Camellia oleifera* cake-protein compound hydrolysate were prepared.

### 2.3. Preparation of Camellia oleifera Cake-Protein Adhesives

First, 12% of crosslinker (E44-epoxy resin) was added into the above obtained pure *Camellia oleifera* cake-protein hydrolysate and *Camellia oleifera* cake-protein compound hydrolysates, and stirred evenly. After standing for 10 min, the pure *Camellia oleifera* cake-protein adhesive (Adhes-1), *Camellia oleifera* cake-protein adhesive modified with DSP (Adhes-2), *Camellia oleifera* cake-protein adhesive modified with SPI (Adhes-3), and *Camellia oleifera* cake-protein adhesive modified with casein (Adhes-4) were prepared, and the formulation of adhesives is shown in Table 1. The pure *Camellia oleifera* cake-protein hydrolysate was taken as the control group (Control). In accordance with the national standard GB/T 14074–2006, the viscosity of adhesives was determined using an NDJ-1 rotary viscometer from Flora Automation Technology Co., Ltd. (Tianjin, China) with a 4# rotor at the speed of 60 r/min.

### 2.4. Preparation of Plywood and the Test of Bonding Strength

Three-layer *Populus* spp. plywood with the dimensions of 400 mm × 400 mm × 5 mm was prepared in the laboratory using the prepared adhesives, through the hot-pressing process (hot-pressing temperature: 160 °C, hot-pressing pressure: 1.0 MPa, hot-pressing time: 5 min, and rate of spreading: 220 g/m^2^ (double-faced, based on liquid adhesives)). The plywood was cut into samples with a size of 100 mm × 25 mm. Next, a wet process-based shear strength test was performed on the plywood samples after the water treatment at 63 ± 3 °C. The shear strength of plywood samples was determined using WDS-50 KN mechanical testing machine produced by Lugong Precision Instrument Co., Ltd. (Kunshan, China), and the final shear strength was the mean value of 8–10 samples.

### 2.5. Insoluble Rate of Cured Adhesives

Each sample was placed into tinfoil and dried in a 120 °C thermostatic drying oven from Niyue Instrument Co., Ltd. (Shanghai, China). Then, it was taken out and ground in a grinding machine, passed through a 200-mesh screen, and sample flours were acquired. The sample flours with the mass of *m*_1_ were wrapped using filter paper and soaked in water at 63 °C for 6 h. Afterwards, they were dried in a 120 °C thermostatic drying oven, and the flour mass was calculated as *m*_2_. The insoluble content in adhesive curing products was calculated according to Equation (1). Each sample was tested for 5 times, and the mean value of insoluble content in the adhesive curing products was taken.

(1) $$\mathrm{Insoluble}\;\mathrm{rate}\;(\%)=\frac{m_{2}}{m_{1}}\;\times \;100\%$$

where *m*_1_ is dry weight of sample before water immersion and *m*_2_ is dry weight of sample after water immersion.

### 2.6. Fourier Transform-Infrared Spectroscopic (FT-IR) Analysis

To explore the influences of DSP, SPI, and casein on the degradation product structure of *Camellia oleifera* cake-protein, an FT-IR test was performed on different *Camellia oleifera* cake-protein hydrolysates using a Varian 1000 (Varian, USA) IR spectrometer under the following parameter conditions: The range of wave number (WN) was 400–4000 cm^−1^, the resolution was 4 cm^−1^, the number of scans was 32, the indoor temperature was 22–25 °C, and the relative humidity was ≤60%.

### 2.7. Differential Scanning Calorimetric (DSC) Analysis

To study the influences of DSP, SPI, and casein on the curing performance of *Camellia oleifera* cake-protein adhesives, a DSC test was implemented using a DSC 204 F1 differential scanning calorimeter produced by the German NETZSCH Company (Rodgau, Germany) with NETZSCH Proteus analysis software under N_2_ protection, along with the following conditions: The range of test temperature was 30–180 °C, and the heating rate was 10 °C/min. Before the test, the adhesive samples were frozen and dried.

### 2.8. Thermogravimetric (TG) Analysis

To evaluate the influences of DSP, SPI, and casein on the thermal properties of *Camellia oleifera* cake-protein adhesives, the cured adhesive was ground into flours for the TG test using a TG 209 F3 thermogravimeter produced by the German NETZSCH Company (Bavaria, Germany) under N_2_ protection, along with the following conditions: The range of test temperature was 30–800 °C, and the heating rate was 10 °C/min.

### 2.9. X-ray Diffraction (XRD) Analysis

To evaluate the influences of DSP, SPI, and casein on the crystallization properties of *Camellia oleifera* cake-protein adhesives, the cured adhesive was ground into flours for the XRD test using a TTR XRD produced by RIGAKU (Tokyo, Japan) under the Cu target (λ = 0.154060 Å), along with the following conditions: The 2θ scanning range was 5–80°, the step size was 0.02°, the scanning rate was 5°/min, the tube current was 120 mA, and the tube voltage was 40 kV.

### 2.10. Scanning Electron Microscopic (SEM) Analysis

Fractured cross-sections of the cured adhesives were analyzed using an S-3400N SEM produced by Hitachi (Tokyo, Japan) at the acceleration voltage of 12.5 kV. The section was observed after metal spraying.

## 3. Results and Discussion

### 3.1. FT-IR Analysis of Camellia oleifera Cake-Protein Hydrolysates

**Figure 1:**
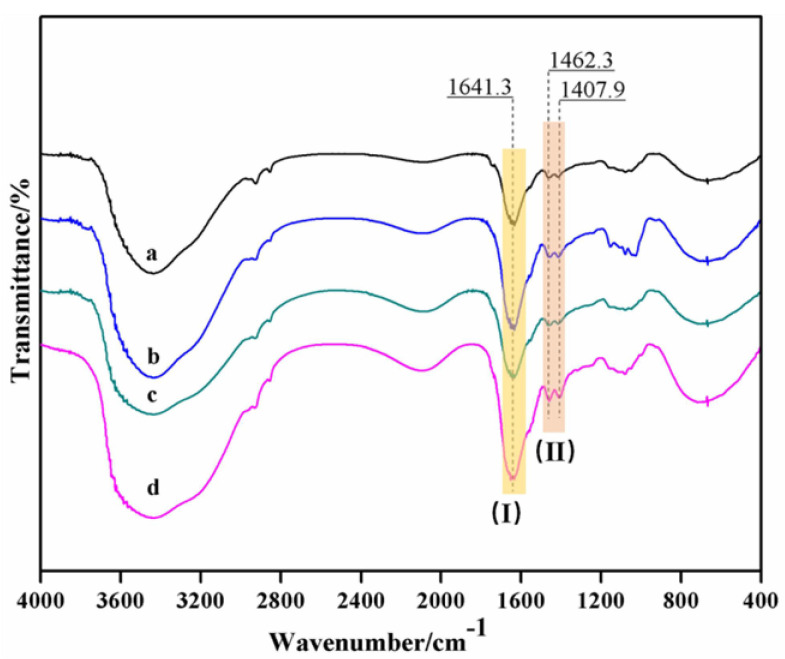
FT-IR spectrogram of *Camellia oleifera* cake-protein hydrolysates. Note: a. *Camellia oleifera* cake-protein hydrolysate; b. SPI/*Camellia oleifera* cake-protein compound hydrolysate; c. DSP/*Camellia oleifera* cake-protein compound hydrolysate; d. Casein/*Camellia oleifera* cake-protein compound hydrolysate; I. amide regions-I; II. amide regions-II.

The FT-IR spectrograms of *Camellia oleifera* cake-protein hydrolysates are displayed in Figure 1.

It was revealed that the pure *Camellia oleifera* cake-protein hydrolysate showed basically consistent IR spectral variation trends with compound hydrolysates, but they differed in the peak intensity, which was mainly manifested in amide regions I (1641.3 cm^−^^1^) and II (1462.3 cm^−^^1^ and 1407.9 cm^−^^1^). The peak of pure *Camellia oleifera* cake-protein hydrolysate was weak in both amide regions I and II, which was ascribed to the relatively lower protein content of *Camellia oleifera* cake. During the degradation process, the accessibility and solubility of alkali contacting *Camellia oleifera* cake-protein were influenced by impurities, such as celluloses; thus, impeding its effective degradation [18,32,33].

After DSP, SPI, and casein were introduced, the peak of amide regions I and II of the degradation products were obviously enhanced, indicating the increasing content of terminal peptide bonds in the system. The possible reasons are described as follows: (1) The total protein content was elevated in the system; (2) The protein introduced experienced alkaline degradation, so the characteristic peaks of the amide regions I and II were enhanced; (3) The alkaline degradation of protein was a chemical equilibrium reaction, and the protein introduced could facilitate the forward degradation reaction of *Camellia oleifera* cake-protein. In addition, the degree of system degradation also varied with DSP, SPI, and casein, where SPI and casein could more easily promote the system degradation, which might be related to the corresponding protein content and molecular weight.

Therefore, the introduction of other proteins promoted the degradation of *Camellia oleifera* cake-protein. The active groups, such as amino and carboxy, generated by the degradation rendered the requisite conditions for the follow-up crosslinking reaction.

### 3.2. Curing Performance of Camellia oleifera Cake-Protein Adhesives

**Figure 2:**
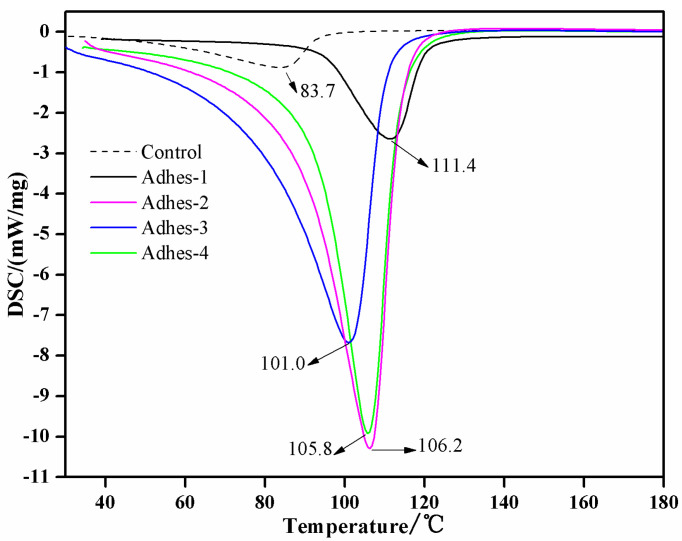
DSC results of *Camellia oleifera* cake-protein adhesives. Note: Control, pure *Camellia oleifera* cake-protein hydrolysate; Adhes-1, the pure *Camellia oleifera* cake-protein adhesive; Adhes-2, Camellia *oleifera* cake-protein adhesive modified with DSP; Adhes-3, *Camellia oleifera* cake-protein adhesive modified with SPI; Adhes-4, *Camellia oleifera* cake-protein adhesive modified with casein.

The DSC results of *Camellia oleifera* cake-protein adhesives are exhibited in Figure 2. It can be seen from Figure 2 that the pure *Camellia oleifera* cake-protein hydrolysate did not have any obvious exothermic peak in the high-temperature (>100 °C) region, only having a very small peak in the low-temperature region (83.7 °C), which was because the pure *Camellia oleifera* cake-protein hydrolysate could not experience a crosslinking reaction, and the curing process mainly involved the intertwining and degradation of protein molecules and the formation of hydrogen bonds. The peak here was mainly induced by the fracture of disulfide bonds in the protein molecules [34,35].

**Figure 3:**
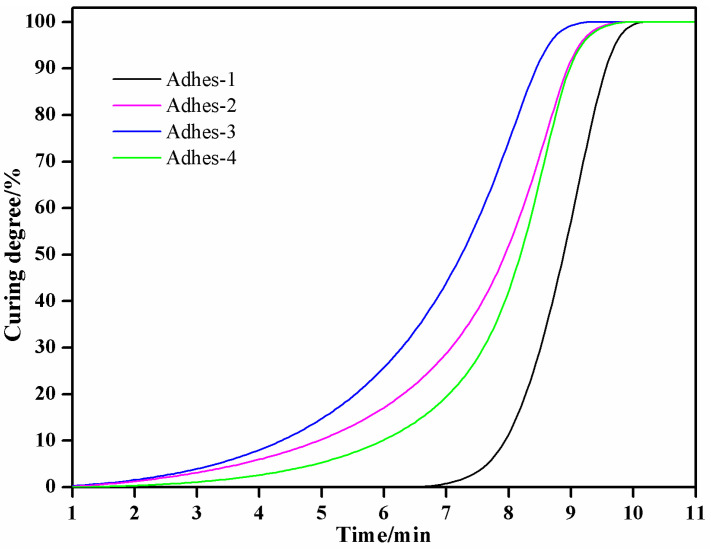
Degree of crosslinking reaction of *Camellia oleifera* cake-protein adhesives. Note: Adhes-1, the pure *Camellia oleifera* cake-protein adhesive; Adhes-2, *Camellia oleifera* cake-protein adhesive modified with DSP; Adhes-3, *Camellia oleifera* cake-protein adhesive modified with SPI; Adhes-4, *Camellia oleifera* cake-protein adhesive modified with casein.

Integral processing was performed for the curing peaks of DSC curves in Figure 2. The curing degree curves (Figure 3) of different *Camellia oleifera* cake-protein adhesives could be obtained by taking curing temperature as the horizontal axis and integral area as the vertical axis. It can be seen from Figure 3 that the curing reaction rate was ordered as follows: pure *Camellia oleifera* cake-protein adhesive < casein-modified *Camellia oleifera* cake-protein adhesive ≤ DSP-modified *Camellia oleifera* cake-protein adhesive < SPI-modified *Camellia oleifera* cake-protein adhesive. Hence, the following results could be acquired: (1) The curing reaction rate of *Camellia oleifera* cake-protein adhesive could be generally improved by introducing defatted soybean protein, SPI, and casein; (2) Due to the differences in protein structure and content, DSP-, SPI-, and casein-modified *Camellia oleifera* cake-protein adhesives were different in curing efficiency, where SPI had the highest efficiency, followed by DSP and casein, successively.

When the pure *Camellia oleifera* cake-protein hydrolysate was used in cooperation with a crosslinker, an evident crosslinking reaction-induced exothermic peak appeared at 111.4 °C. On this basis, obvious curing exothermic peaks also occurred with DSP-, SPI-, and casein-modified *Camellia oleifera* cake-protein adhesives. By comparing the DSC results in Figure 2, the following results were obtained:

(1) The crosslinking reaction temperature between pure *Camellia oleifera* cake-protein hydrolysate and crosslinker was the highest, with the lowest heat release, indicating the low reactivity of pure *Camellia oleifera* cake-protein hydrolysate, its unideal reaction with the crosslinker, and a low crosslinking reaction degree.
(2) The crosslinking reaction between *Camellia oleifera* cake-protein compound hydrolysate and crosslinker moved towards a low temperature, and the heat release was significantly increased, showing that the reactivity of the hydrolysate was significantly enhanced by introducing other proteins, which facilitated sufficient reaction with the crosslinker and reached a high degree of crosslinking reaction.
(3) DSP-, SPI-, and casein-modified *Camellia oleifera* cake-protein adhesives all showed significant crosslinking reaction-induced exothermic peaks, but their peak temperature and heat release were different. The peak curing temperature of SPI-modified *Camellia oleifera* cake-protein adhesive (101.0 °C) < that of casein-modified *Camellia oleifera* cake-protein adhesive (105.8 °C) < that of DSP-modified *Camellia oleifera* cake-protein adhesive (106.2 °C).

Curing temperature was associated with the active site and apparent activation energy of hydrolysates. The SPI-modified *Camellia oleifera* cake-protein adhesive reached the minimum curing temperature and a low apparent activity energy, reflecting that there were many active functional groups and active sites within the unit volume of compound hydrolysates at the time. A possible reason was that the high protein content of SPI could more easily promote the degradation of *Camellia oleifera* cake-protein, and, thus, more active functional groups were exposed and the active sites for the reaction with the crosslinker were increased. Different protein-modified *Camellia oleifera* cake-protein adhesives had different curing temperatures, which was correlated with the protein category and protein content. Moreover, they also differed in promoting the degradation of *Camellia oleifera* cake-protein. Meanwhile, the results demonstrated that the hot-pressing temperature 160 °C experimentally adopted was reasonable.

### 3.3. SEM Analysis

**Figure 4:**
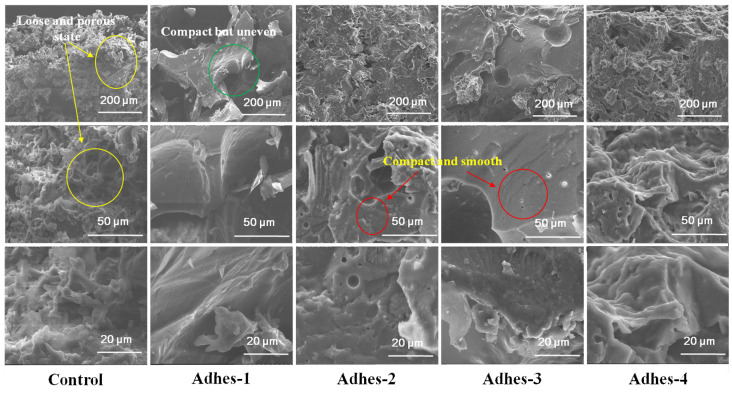
SEM images of fractured cross-sections of cured *Camellia oleifera* cake-protein adhesives. Note: Control, pure *Camellia oleifera* cake-protein hydrolysate; Adhes-1, the pure *Camellia oleifera* cake-protein adhesive; Adhes-2, Camellia *oleifera* cake-protein adhesive modified with DSP; Adhes-3, *Camellia oleifera* cake-protein adhesive modified with SPI; Adhes-4, *Camellia oleifera* cake-protein adhesive modified with casein.

Figure 4 shows the SEM images of fractured cross-sections of cured *Camellia oleifera* cake-protein adhesives. It can be seen by combining the DSC results that the pure *Camellia oleifera* cake-protein hydrolysate itself could not experience a crosslinking reaction. Due to the vapor volatilization in the curing process, the section of adhesive layer presented a disorderly, loose, and porous state, indicating its low cohesion and high brittleness [36]. Meanwhile, holes, which served as water channels, reduced the water resistance of the adhesives.

When the pure *Camellia oleifera* cake-protein hydrolysate was utilized in cooperation with the crosslinker, the sectional structure of the adhesive layer started to become compact and uneven, showing that a crosslinking reaction took place in the curing process, and the cohesion started to increase. However, the crosslinking reaction was uneven, due to the unevenness of the *Camellia oleifera* cake-protein hydrolysate.

When the *Camellia oleifera* cake-protein compound hydrolysate was used together with the crosslinker, the surface of the loose adhesive layer was obviously improved, being compact and smooth with increasing wrinkles, and this showed that the cohesion and crosslinking density of the adhesive were remarkably enhanced. This was because DSP, SPI, and casein facilitated the degradation of the *Camellia oleifera* cake-protein, and more active groups were generated in the system; thus, promoting the reaction with the crosslinker. The cured adhesive layer formed a complex crosslinked spatial reticular system, with a high cohesion and strong water resistance. In particular, the cross section of the adhesive layer in the DSP- and SPI-modified *Camellia oleifera* cake-protein adhesives was the smoothest, meaning that the crosslinking reaction was relatively radical, which accorded with the DSC analysis results.

### 3.4. Heat Resistance of Camellia oleifera Cake-Protein Adhesives

**Figure 5:**
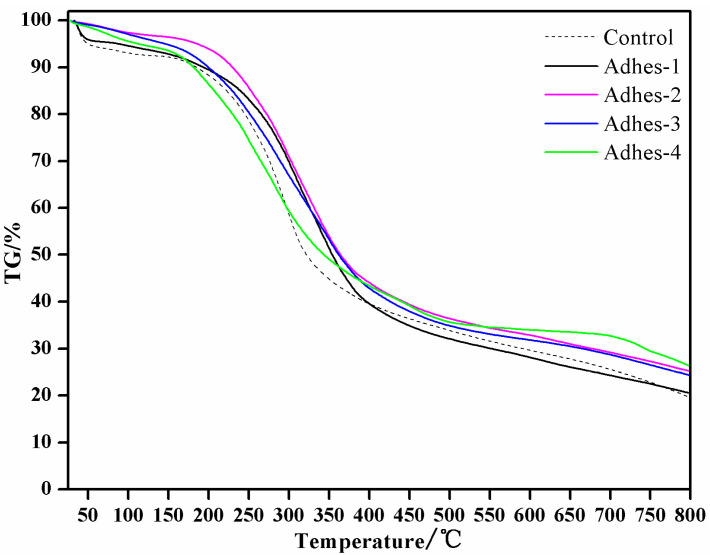
TG results of *Camellia oleifera* cake-protein adhesives. Note: Control, pure *Camellia oleifera* cake-protein hydrolysate; Adhes-1, the pure *Camellia oleifera* cake-protein adhesive; Adhes-2, Camellia *oleifera* cake-protein adhesive modified with DSP; Adhes-3, *Camellia oleifera* cake-protein adhesive modified with SPI; Adhes-4, *Camellia oleifera* cake-protein adhesive modified with casein.

**Figure 6:**
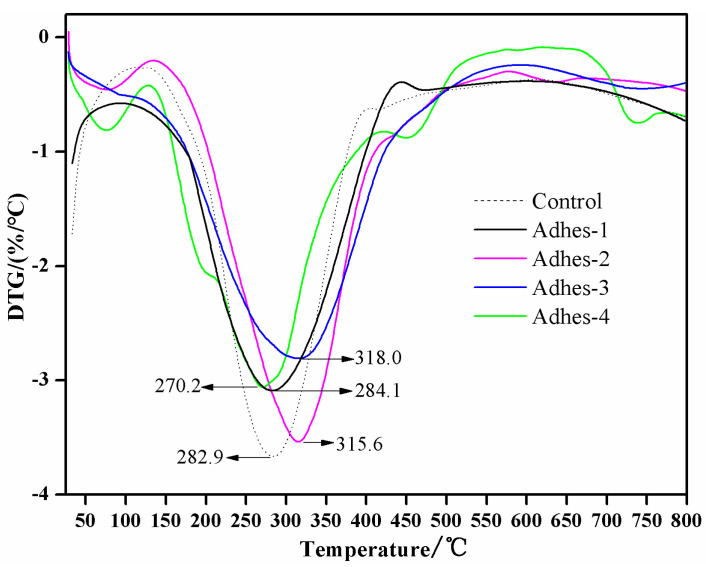
DTG results of *Camellia oleifera* cake-protein adhesives. Note: Control, pure *Camellia oleifera* cake-protein hydrolysate; Adhes-1, the pure *Camellia oleifera* cake-protein adhesive, Adhes-2, Camellia *oleifera* cake-protein adhesive modified with DSP; Adhes-3, *Camellia oleifera* cake-protein adhesive modified with SPI; Adhes-4, *Camellia oleifera* cake-protein adhesive modified with casein.

The TG and derivative TG (DTG) results of *Camellia oleifera* cake-protein adhesives are exhibited in Figure 5 and Figure 6. The thermolysis of the *Camellia oleifera* cake-protein adhesives could be divided into four stages: The first stage was 50–130 °C, in which adhesive curing product’s absorbed water evaporated in the air and the mass loss was generally within 10%. The weight loss was the highest in the curing products of pure *Camellia oleifera* cake-protein hydrolysate, which was associated with the loose and porous state on the cross section of its adhesive layer without crosslinking reaction. The second stage was 130–200 °C, in which the fracture of the protein secondary structure dominated. The third stage was 200–500 °C, in which the mass loss rate was the highest, accompanied by the degradation of protein peptide bonds and the skeleton structure of the adhesive. Before and after the crosslinker was added to the pure *Camellia oleifera* cake-protein hydrolysate, the temperature leading to the maximum weight loss rate was 282.9 °C and 284.1 °C, respectively; further indicating that the *Camellia oleifera* cake-protein hydrolysate experienced a crosslinking reaction with the crosslinker; thus, generating relatively stable crosslinked products. The temperature triggering the maximum weight loss rate of curing products of DSP- and SPI-modified *Camellia oleifera* cake-protein adhesives rose to 315.6 °C and 318 °C, respectively, showing that the thermal stability of the adhesive was enhanced with the introduction of DSP and SPI. The temperature leading to the maximum weight loss rate of curing products of casein/*Camellia oleifera* cake compound adhesive shifted to a lower temperature 270.2 °C, meaning that the thermal stability of the adhesive was degraded by the casein being introduced.

### 3.5. XRD Analysis

**Figure 7:**
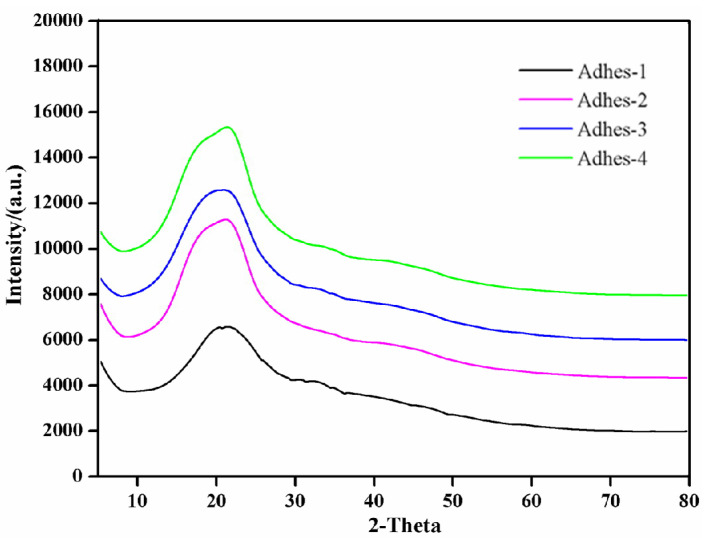
XRD results of curing products of *Camellia oleifera* cake-protein adhesives. Note: Adhes-1, the pure *Camellia oleifera* cake-protein adhesive; Adhes-2, Camellia *oleifera* cake-protein adhesive modified with DSP; Adhes-3, *Camellia oleifera* cake-protein adhesive modified with SPI; Adhes-4, *Camellia oleifera* cake-protein adhesive modified with casein.

The XRD results of the cured *Camellia oleifera* cake-protein adhesives are displayed in Figure 7. The degree of crystallinity denoted the degree of ordered molecular arrangement. The degree of crystallinity in the products of the crosslinking reaction between pure *Camellia oleifera* cake-protein hydrolysate and crosslinker was relatively low, which indicated a low degree of crosslinking reaction. After the *Camellia oleifera* cake-protein compound hydrolysate experienced the crosslinking reaction with the crosslinker, the degree of crystallinity was evidently elevated, which showed a high degree of crosslinking reaction. As further proved by the XRD results, a crosslinked structure was established in the *Camellia oleifera* cake-protein adhesive, and its crosslinking density could be further improved by introducing DSP, SPI, and casein.

### 3.6. Insolubility Rate in the Cured Camellia oleifera Cake-Protein Adhesives

**Figure 8:**
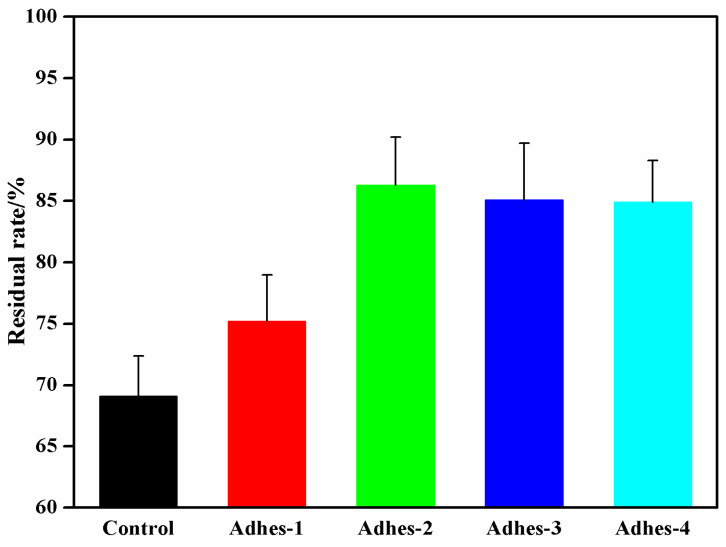
Insoluble rate of cured *Camellia oleifera* cake-protein adhesives. Note: Control, pure *Camellia oleifera* cake-protein hydrolysate; Adhes-1, the pure *Camellia oleifera* cake-protein adhesive; Adhes-2, Camellia *oleifera* cake-protein adhesive modified with DSP; Adhes-3, *Camellia oleifera* cake-protein adhesive modified with SPI; Adhes-4, *Camellia oleifera* cake-protein adhesive modified with casein.

The results regarding the proportion of insoluble matters in the curing products of *Camellia oleifera* cake-protein adhesives are seen in Figure 8. The insolubility rate in the curing products before and after the pure *Camellia oleifera* cake-protein hydrolysate was added with the crosslinker was 69.1% and 75.2%, respectively. In the former case, no crosslinking reaction occurred, but the protein molecules were intertwined in the curing process, with a certain water resistance within the experiment. However, the water resistance was relatively low, since the curing layer was loose and porous, which let water molecules in easily. In the latter case, a certain crosslinking reaction took place and a certain number of water-resistant crosslinking products were generated, but the insolubility rate was only improved by 8.8% due to the lack of high crosslinking density.

The insolubility rate in the cured products of DSP-, SPI- and casein-modified *Camellia oleifera* cake-protein adhesives was 85.1%, 86.3%, and 84.9%, respectively; with an increase in value of 24.9%, 23.2%, and 22.9%, respectively. The insoluble rate could also be used to qualitatively characterize the crosslinking density and water resistance of the adhesives. It was shown by combining the FTIR, DSC, DTG, and SEM results that DSP, SPI, and casein facilitated the hydrolysis of *Camellia oleifera* cake-protein and increased the active sites for the reaction, which contributed to sufficient crosslinking reaction with the crosslinker, the improved crosslinking density of curing products, and are markably enhanced insolubility rate.

### 3.7. Basic Properties of Camellia oleifera Cake-Protein Adhesives

The results of the viscosity and storage time of *Camellia oleifera* cake-protein adhesives are listed in Table 2. Before and after the pure *Camellia oleifera* cake-protein hydrolysate was added to the crosslinker, the viscosity was 21.6 and 28.5 mPa·s, respectively, and the adhesive solution was laminated in both cases, indicating that the pure *Camellia oleifera* cake-protein could not be effectively degraded. On the one hand, this affected the degree of follow-up crosslinking reaction, and on the other hand, it influenced the adhesive storage stability. In the DSP-, SPI-, and casein-modified *Camellia oleifera* cake-protein adhesives, the adhesive solution was even, with the viscosity significantly increased and the storage time remarkably shortened, indicating that the crosslinked structure and crosslinking density of the *Camellia oleifera* cake-protein adhesive were further strengthened. In addition, the viscosity of the casein-modified *Camellia oleifera* cake-protein adhesive was excessively increased due to the high reactivity of casein, which impeded the diffusion, wetting, and permeation of the adhesive on the wood surface and further impacted the bonding performance.

**Figure 9:**
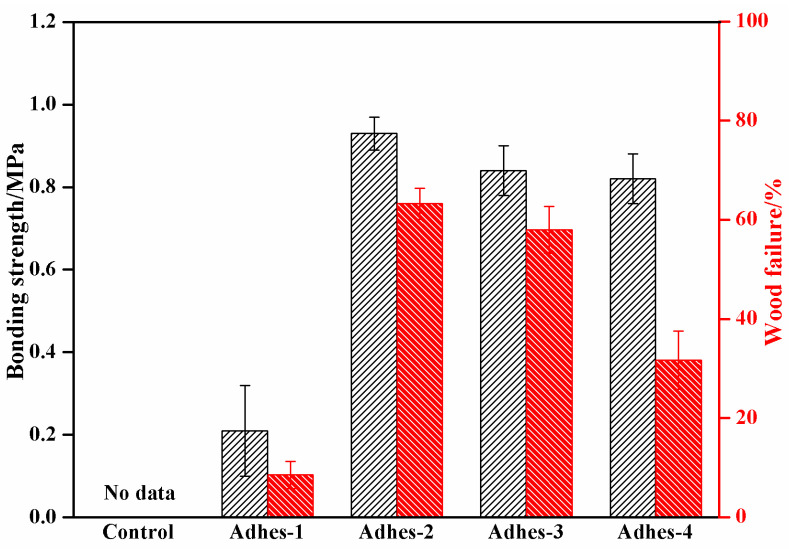
Results of bonding strength and wood failure of *Camellia oleifera* cake-protein adhesives. Note: Control, pure *Camellia oleifera* cake-protein hydrolysate; Adhes-1, the pure *Camellia oleifera* cake-protein adhesive; Adhes-2, Camellia *oleifera* cake-protein adhesive modified with DSP; Adhes-3, *Camellia oleifera* cake-protein adhesive modified with SPI; Adhes-4, *Camellia oleifera* cake-protein adhesive modified with casein.

Regarding bonding, wood failure is bound to occur at the weakest part of the bonded object, through the complex physical and chemical reactions between the adhesive and the bonded object. Assuming that the failure occurs at the adhesive layer, the wood failure should be 0%. If the bonding is effective, the wood failure rate should be 100%. Therefore, wood failure certainly occurs at the weakest part of the wood. However, contradictorily, the corresponding strength is not the real bonding strength of the wood, but the strength at its weakest part. In other words, the bonding strength in real terms means that the wood and adhesive layer simultaneously exists on the shear section. Hence, wood failure is still required to evaluate the bonding reliability. The test results of shear strength and wood failure of plywood prepared using *Camellia oleifera* cake-protein adhesives are exhibited in Figure 9. The pure *Camellia oleifera* cake-protein hydrolysate did not experience any crosslinking reaction; hence, it was without any wet strength. The plywood sample was directly boiled open, without a wood failure rate. After the crosslinker was added, the plywood showed a certain wet bonding strength (0.21 MPa) due to the established crosslinked structure, and the wood failure rate was only 8%. In this case, both the bonding strength and wood failure rate were the lowest, and the standard deviation of bonding strength was the maximum, showing the unstable plywood performance. It was found in the experimental process that the adhesive prepared using the pure *Camellia oleifera* cake-protein hydrolysate had a poor initial viscosity and evenness, along with easy delamination. Based on previous experience, this was attributed to the poor sizing performance, resulting from the large viscosity. However, the viscosity of the adhesive was small (28.5 mPa·s) under this circumstance, so the unstable plywood performance was more likely derived from the uneven degradation of *Camellia oleifera* cake, as well as the degree of follow-up crosslinking reaction and the uneven molecular weights of reaction products, and so on.

The bonding process of adhesives refers to their flow, wetting, permeation, and curing process on the wood surface until forming glue nails. Wood is a porous material, containing large capillary voids and microcapillary voids. Under the action of hot-pressing temperature and pressure, the adhesive will continuously permeate into these voids and finally be cured, to form firm glue nails. In this study, however, the casein-modified *Camellia oleifera* cake-protein adhesive showed a high viscosity, so its mobility was poor, and due to the insufficient permeation of the adhesive, no good glue nails could be formed, which contributed to a relatively low wood failure rate.

### 3.8. Reinforcement Mechanism Analysis of Camellia oleifera Cake-Protein Adhesives

The *Camellia oleifera* cake-protein content was relatively low. Impurities such as celluloses in the degradation process influenced the accessibility and solubility of alkali contacting the *Camellia oleifera* cake-protein and impacted the degradation of the *Camellia oleifera* cake-protein and the subsequent crosslinking reaction. Consequently, the prepared adhesive presented a poor initial viscosity and evenness, and it was prone to delamination.

**Figure 10:**
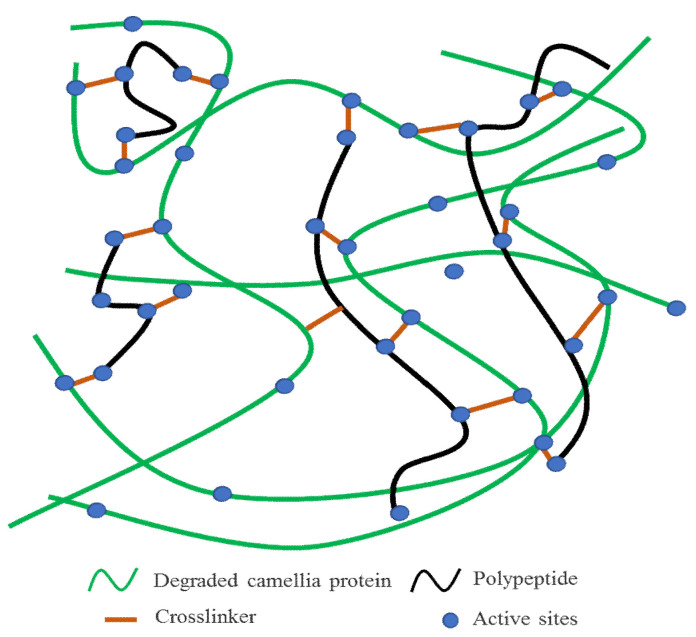
Schematic diagram of peptide chain reinforced *Camellia oleifera* cake-protein adhesives.

From the perspective of chemical equilibrium, the introduced DSP, SPI, and casein promoted the effective degradation of *Camellia oleifera* cake-protein, so that the protein was more prone to degradation, dispersion, and extension in the alkaline solution: (1) More reactivity sites were generated in the system (Figure 10), which was good for the bonding of the adhesive in the chemical form, and hydrogen bonds were transformed into covalent bonds, which was more conducive to the establishment of a compact crosslinked structure [36,37]; (2) DSP, SPI, and casein were degraded into peptide chains with lower molecular weights, which were interspersed in the system; thus, enhancing the overall flexibility of the adhesive and enhancing the acting force and hydrogen-bond interaction between the crosslinker and hydrolysate. Moreover, this contributed to a better elastic contact and regular array between crosslinking products; the crosslinked structure and crosslinking density were further strengthened (Figure 10); and the cured adhesive layer formed a compact reticular structure, which could prevent the wedging of moisture and further strengthened the water resistance of the adhesive; (3) The toughening effect of peptide chains relieved the increase in the brittleness induced by the excessive crosslinking of the adhesive system and the water evaporation in the hot-pressing process, and avoided the degradation of the bonding performance, which was macroscopically shown by the good bonding performance and durability; (4) In addition, the branching of the adhesive system enlarged the contact area with the fibrous structure of the wood, enhanced the interaction between hydrophilic groups and hydrophobic groups, and further improved the bonding performance of the *Camellia oleifera* cake-protein adhesives.

## 4. Conclusions

In this study, *Camellia oleifera* cake flours were blended with defatted soybean protein (DSP), soybean protein isolate (SPI), and casein, to prepare adhesives through the degradation and crosslinking methods. The results showed that:

(1) DSP, SPI, and casein likely promoted the effective degradation of *Camellia oleifera* cake-protein; more active groups and reactivity sites were formed in the system; the initial viscosity and stability of the adhesive were reinforced, but its usable life was shortened.
(2) DSP-, SPI-, and casein-modified *Camellia oleifera* cake-protein adhesives showed a lower curing reaction temperature and significantly increased heat release. SPI was more inclined to facilitate the degradation of *Camellia oleifera* cake-protein, so more active functional groups were exposed, and the active sites for the reaction with the crosslinker were increased, which contributed to the establishment of a compact crosslinked structure.
(3) DSP, SPI, and casein, themselves, could be degraded into peptide chains with lower molecular weights, which improved the overall flexibility of the adhesive, boosted the elastic contact and regular array between the crosslinking products, and further enhanced the crosslinked structure and crosslinking density. The cured adhesive layer formed a compact reticular structure, with both bonding strength and water resistance being notably improved.
(4) Based on the bonding performance, storage time and initial viscosity, the next step will be to adopt for study the DSP-modified *Camellia oleifera* cake-protein adhesive, mainly studying the amount of DSP, the amount of crosslinker, and the hot pressing process. Moreover, the results can also provide a reference for the development of other oil cake-protein adhesives.

## Figures and Tables

**Table 1:** The formulation of adhesives.

Adhesives	Cake Protein/g	DSP /g	SPI /g	Casein /g	SDBS /g	Urea /g	NaOH /g	Crosslinker /%
Control	80	-	-	-	1.2	8	4.8	-
Adhes-1	80	-	-	-	1.2	8	4.8	12
Adhes-2	72	8	-	-	1.2	8	4.8	12
Adhes-3	72	-	8	-	1.2	8	4.8	12
Adhes-4	72	-	-	8	1.2	8	4.8	12

**Table 2:** Viscosity and storage time of *Camellia oleifera* cake-protein adhesives.

Adhesives	Viscosity/mPa·s	Storage Time/h	Remark
Control	21.6 (±2.2)	72 (±3.5)	Adhesive solution delaminated
Adhes-1	28.5 (±2.5)	10 (±0.6)	Adhesive solution delaminated
Adhes-2	108.8 (±15)	3 (±0.3)	Even adhesive solution
Adhes-3	115.0 (±13)	2 (±0.3)	Even adhesive solution
Adhes-4	7698.3 (±364)	1 (±0.2)	Even adhesive solution

Note: Control, pure *Camellia oleifera* cake-protein hydrolysate; Adhes-1, the pure *Camellia oleifera* cake-protein adhesive; Adhes-2, Camellia *oleifera* cake-protein adhesive modified with DSP; Adhes-3, *Camellia oleifera* cake-protein adhesive modified with SPI; Adhes-4, *Camellia oleifera* cake-protein adhesive modified with casein.

## Data Availability

Data sharing is not applicable to this article.
